# Acute fluoroacetamide poisoning with reversible leukoencephalopathy: A case report and literature review highlighting the effectiveness of acetamide and the diagnostic value of neuroimaging

**DOI:** 10.1097/MD.0000000000048732

**Published:** 2026-05-15

**Authors:** Zhongliang Xu, Shaopeng Ma, Zhuangfei Li, Shengkun Yang, Deyu Yang, Zhengze Shen, Shudong Liu

**Affiliations:** a Department of Pharmacy, The Affiliated Yongchuan Hospital of Chongqing Medical University, Chongqing Municipal, P. R. China; b Department of Neurology, The Affiliated Yongchuan Hospital of Chongqing Medical University, Chongqing Municipal, P. R. China; c Department of Pharmacy, Maternal and Child Health Hospital of Yongchuan District, Chongqing Municipal, P. R. China; d Department of Neurology, Sanjiao Town Health Center of Yongchuan District, Chongqing Municipal, P. R. China.

**Keywords:** acetamide, fluoroacetamide, fluoroacetic acid derivatives, magnetic resonance imaging, reversible leukoencephalopathy

## Abstract

**Rationale:**

Acute fluoroacetamide poisoning is a rare and life-threatening emergency. Diagnosis is challenging due to nonspecific symptoms and a latent period. This report emphasizes the effectiveness of delayed acetamide treatment and the importance of neuroimaging for diagnosis and monitoring.

**Patient concerns:**

A 58-year-old man was admitted to the emergency room about 5 hours after intentionally ingesting an unknown pesticide following a personal conflict.

**Diagnoses:**

Fluoroacetamide poisoning was initially suspected based on prior domestic case reports, the patient history of pesticide ingestion, clinical signs, and diffusion-weighted magnetic resonance imaging (MRI) showing characteristic features of cytotoxic edema in specific brain regions. Upon confirming the pesticide product with the seller, the diagnosis was ultimately confirmed as fluoroacetamide poisoning.

**Interventions:**

Gastric lavage, intramuscular acetamide, fluid resuscitation, and electrolyte correction were administered.

**Outcomes:**

The patient showed rapid clinical improvement after receiving acetamide. He was discharged after one week without any additional medication. Follow-up MRI at one month showed complete resolution of the previous abnormalities, and he reported no symptoms at the one-year follow-up.

**Lessons:**

This case highlights the potential advantages of delaying acetamide treatment and suggests that brain diffusion-weighted MRI could be valuable in diagnosing and monitoring acute fluoroacetamide poisoning.

## 1. Introduction

Pesticide poisoning represents a critical public health issue worldwide.^[1]^ Fluoroacetamide, a highly toxic fluorinated organic compound originally designated as Compound 1081, was developed in the mid-20th century as a potent rodenticide and insecticide. It is classified by the World Health Organization as a Category Ib hazardous agent due to its acute oral toxicity. Although banned in many countries, including China since the 1970s, its low cost and chemical stability contribute to its continued illegal use, especially in some developing regions, resulting in repeated cases of human poisoning.^[2,3]^

The toxicity of fluoroacetamide stems from its conversion to fluoroacetate, which disrupts the tricarboxylic acid cycle by inhibiting aconitase, ultimately leading to severe energy depletion.^[4]^ Tissues with high metabolic demands, such as the brain and heart, are predominantly affected, resulting in nonspecific but severe symptoms including impaired consciousness, seizures, and cardiac arrhythmias.^[5–7]^ These symptoms often resemble those of other acute neurological or cardiac conditions, which makes prompt diagnosis challenging.^[8]^

This case report discusses a patient with acute fluoroacetamide poisoning who showed reversible leukoencephalopathy on magnetic resonance imaging (MRI) and was successfully treated with acetamide antidotal therapy. We also reviewed the literature on poisoning by fluoroacetic acid derivatives, with a focus on treatment protocols and the role of neuroimaging in management and monitoring.

## 2. Case presentation

A 58-year-old man presented to the emergency room about 5 hours after intentionally ingesting a pesticide following an interpersonal conflict. Upon admission, the patient was conscious but unable to confirm what substance had been ingested. His medical history included a pesticide ingestion over 20 years ago, from which he recovered completely, along with chronic alcohol use. There was no significant family history of neurological disease. In the emergency room, gastric lavage was performed. During observation, he developed decreased consciousness, pallor, and no response to orbital pressure, as well as no voluntary limb movements, for approximately 30 minutes. Coagulation profiles were within normal limits.

Due to persistent consciousness disturbance, he was transferred to the neurology department. Neurological examination showed voluntary eye-opening, agitation, and distress to painful stimuli. His gaze was deviated to the right, with pupils equal and reactive at 4.0 mm. The neck was supple, and both Babinski and Brudzinski signs were absent. No hemorrhages or ecchymoses were observed on skin or mucosal examination.

**Figure 1.**
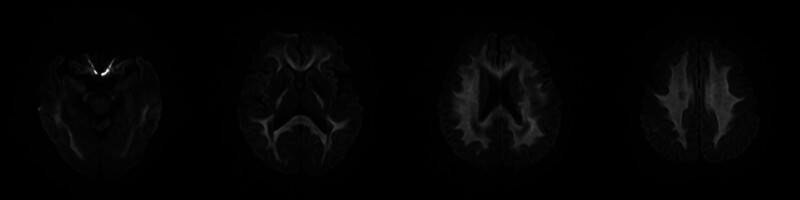
Brain diffusion-weighted MRI during the acute stages of fluoroacetamide intoxication in a 58-year-old man. Abnormal signals in the cerebral peduncles, corpus callosum, internal and external capsules, and cerebral white matter. MRI = magnetic resonance imaging.

Pertinent laboratory findings on admission showed hypokalemia (2.5 mmol/L), elevated creatine kinase (204.0 U/L), and increased troponin (0.11 ng/mL). Gamma-glutamyl transferase (105.0 U/L) and ammonia levels (88.0 μmol/L) were also elevated. Toxicologic screening was declined by the family. Brain diffusion-weighted MRI on the second day of hospitalization demonstrated abnormal signals consistent with cytotoxic edema in the cerebral peduncles, corpus callosum, internal and external capsules, and cerebral white matter (Fig. 1). Based on previous domestic case reports, the patient clinical presentation and radiologic findings, fluoroacetamide poisoning was initially suspected. Acetamide (5.0 g 3 times daily) was administered via intramuscular injection immediately. Supportive care included intravenous fluids and potassium replacement. The diagnosis was confirmed on the third day of hospitalization after the pesticide seller was contacted and identified the product as fluoroacetamide.

**Figure 2.**
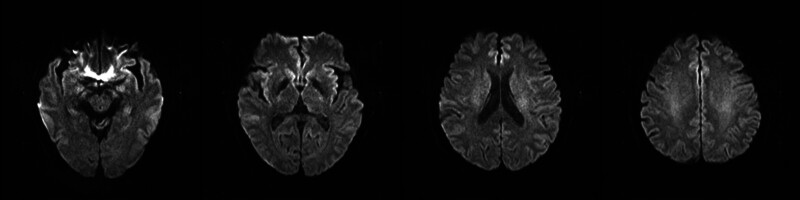
Brain diffusion-weighted MRI were obtained one month later. Note that the abnormal signals shown in Figure 1. Completely disappeared. MRI = magnetic resonance imaging.

The patient showed rapid clinical improvement following acetamide treatment. He was discharged after one week of hospitalization without any additional medication. A follow-up MRI at one month demonstrated complete resolution of the previous abnormalities (Fig. 2). At the one-year follow-up, he remained asymptomatic and expressed gratitude for his recovery, while also expressing regret for his actions.

## 3. Literature review and methods

The study was conducted in accordance with the Declaration of Helsinki and approved by the Medical Research Ethics Committee of Yongchuan Hospital, Chongqing Medical University (approval number 202449). Written informed consent was obtained from the patient for the publication of this case report and accompanying images.

An extensive search of the English-language literature was conducted using the online databases PubMed, Web of Science, and Embase, encompassing all records up to September 15, 2025. The search used various combinations of terms based on the main circulation and usage of fluoroacetic acid derivatives in the past, including fluoroacetamide, fluoroacetate, monofluoroacetate, β-fluoroethyl acetate, and poisoning or intoxication. Only case reports and case series were included; those without full-text access were excluded. Following a thorough review of all identified cases and the available English literature, 13 reports involving 14 cases were included in the final analysis. Neuroimaging findings were detailed in 8 of these cases. A summary of these cases is provided in Table 1

**Table 1:** Characteristics and main findings of fluoroacetic acid derivatives poisoning cases.

Author	Country and yr	Age (yr), sex, and intent	Pesticide	Clinical presentation	Neuroimaging finding	Treatment	Outcome
Gajdusek DC^[9]^	USA, 1950	2, male, accidental	Sodium monofluoroacetate	Vomiting, retching, nausea, emesis, generalized convulsive movements, stupor, cardiac irregularity, apnea	NR	Gavage, calcium gluconate, atropine, amobarbital	Survival
Taitelman U^[5]^	Israel, 1983	17, male, intentional	Fluroacetamide	Vomiting, deep coma, repeated grand mal seizures, arrhythmia	NR	Gastric lavage, activated carbon, sodium pentothal, monoacetin, calcium chloride	Survival
Trabes J^[10]^	Israel, 1983	15, female, intentional	Sodium monofluoroacetate	Nausea, vomiting, abdominal pain, repeated grand mal seizure, tachycardia, profuse sweating, coma	CT: moderate brain atrophy with widening of the basal cisterns, quadrigeminal cistern, interhemispheric fissure, lateral ventricles, and third ventricle	Phenytoin, pentothal, glycerol monoacetate	Survival
Chi CH^[11]^	China, 1999	26, female, intentional	Sodium monofluoroacetate	Nausea, vomiting, hypotension, shock, altered consciousness, respiratory failure	NR	Aggressive inotropic support, fluid supply, electrolyte correction	Death
		62, female, intentional	Sodium monofluoroacetate	Nausea, vomiting, shock, gastrointestinal bleeding, frequent ventricular premature beats	NR	Aggressive inotropic support, fluid supply, electrolyte correction, and antibiotics	Survival
Kim JY^[12]^	South Korea, 2008	32, female, intentional	β-Fluoroethyl acetate	Nausea, vomiting, stuporous condition	MRI: symmetrical hyperintensities of bilateral internal capsules, deep and subcortical white matter, and corpus callosum on DWI	Gastric lavage, activated carbon, conservative treatment	Survival
Im TH^[13]^	South Korea, 2009	32, female, intentional	Sodium monofluoroacetate	Nausea, vomiting, seizure,	MRI: increased signal intensity bilaterally in the cerebellar peduncles, cerebral peduncles, internal capsules, corpus callosum, and corona radiata on DWI	Nasogastric tube suctioning, isosorbide dinitrate, fluid, sedatives, electrolyte replacement therapy	Survival
Kim JB^[14]^	South Korea, 2014	38, male, intentional	Sodium monofluoroacetate	Vomiting and progressive mental deterioration	MRI: extensive white matter restricted diffusion	NR	Survival
Wang R^[8]^	China, 2016	48, male, intentional	Fluroacetamide	Dysphagia, dysphonia, aphasia, generalized convulsions, coma	CT: hypoxic-ischemic changes are seen lightly in the hippocampal regions and the cerebral cortex	NR	Survival
Wen W^[15]^	China, 2017	65, female, NR	Fluroacetamide	Nausea, vomiting, drowsiness, loss of consciousness, coma	NR	Gastric lavage, acetamide, calcium gluconate, vitamin C, and traditional Chinese medicine	Survival
Jin JH^[16]^	South Korea, 2017	62, female, intentional	β-Fluoroethyl acetate	Dizziness, dysarthria, and clumsiness of the hands	MRI: severe cerebellar atrophy, mostly in the cerebellar hemispheres rather than the vermis	NR	Survival
Reyes DA^[17]^	Colombia, 2020	14, female, intentional	Sodium fluoroacetate	Dyspnea, fasciculations, hypotension, altered consciousness, shock	NR	Gastric lavage, activated carbon, ethanol, calcium gluconate, norepinephrine	Survival
Lu A^[18]^	China, 2021	40, male, NR	Fluroacetamide and bromadiolone	Headache, weakness, generalized tonic-clonic seizure, disorder of consciousness, coagulation disorders	MRI: hyperintense lesions throughout the corpus callosum and on both sides of the brachium pontis, posterior limb of the internal capsule, periventricular white matter, centrum semiovale, and corona radiata on DWI	Hemoperfusion, fresh frozen plasma, vitamin K1, diazepam, pantoprazole, sodium valproate, acetamide, calcium gluconate	Survival
Liang S^[19]^	China, 2025	62, female, NR	Fluroacetamide	Vomiting, slurred speech, inappropriate responses, agitation, and generalized tonic-clonic seizure	MRI: high signal lesions on DWI in the subcortical white matter of the bilateral frontoparietal lobes, posterior limbs of the bilateral internal capsules, corpus callosum (genu, body, and splenium), and bilateral cerebral peduncles	Hemoperfusion, vitamin K1, pantoprazole, levetiracetam, acetamide	Survival

CT = computed tomography, DWI = diffusion-weighted imaging, MRI = magnetic resonance imaging, NR = not reported.

## 4. Discussion

The geographic disparity in reported cases of fluoroacetic acid derivatives poisoning reflects different historical and regulatory contexts in various regions. In East Asia, most cases of human poisoning involved compounds that were once common but have now been banned. China has reported numerous cases of fluoroacetamide poisoning,^[20]^ whereas South Korea has reported cases involving β-fluoroethanol derivatives.^[16]^ These cases are usually the result of intentional poisoning or accidental ingestion of contaminated food.

The toxicokinetics of fluoroacetamide involve rapid absorption through the gastrointestinal tract and potential entry via the respiratory mucosa, open wounds, or ocular exposure.^[15]^ The probable lethal oral dose in humans is <5 mg/kg of body weight, or less than 7 drops for a 150-lb. person. A notable feature of fluoroacetamide poisoning is the variable latent period ranging from 30 minutes to several hours, even after lethal exposure,^[6,21]^ which underscores the necessity of observing asymptomatic patients for at least 24 hours. Clinical presentation often includes nausea, vomiting, neurological decline, and cardiac dysfunction, which makes early differentiation from other acute medical conditions challenging.^[5,8,15]^

The pathophysiological mechanism of acute fluoroacetamide poisoning involves the formation of fluorocitrate, which occurs when fluoroacetate condenses with oxaloacetate. Fluorocitrate acts as a potent inhibitor of aconitase, a key enzyme in the tricarboxylic acid cycle.^[4,22,23]^ Astrocytes appear particularly vulnerable due to their high reliance on this cycle for glutamine synthesis and potassium homeostasis, making them susceptible to energy failure and subsequent cytotoxic edema.^[22]^

Neuroimaging, particularly diffusion-weighted MRI, plays a valuable role in diagnosis. It detects cytotoxic edema, an early marker of energy failure that often manifests as symmetric hyperintensities in the cerebral white matter, corpus callosum, internal capsule, and brainstem structures.^[13,14,18]^ As demonstrated in our case and supported by the literature on fluoroacetamide, sodium monofluoroacetate, and β-Fluoroethyl acetate poisoning, these radiographic findings are often reversible with clinical recovery, reinforcing the idea of a dynamic and potentially treatable leukoencephalopathy.^[12–14,16,18]^ The pathophysiological basis involves fluoroacetate-induced inhibition of the glial Krebs cycle, leading to transient energy failure without immediate irreversible damage.^[22]^

Treatment remains primarily supportive, including gastric lavage, activated charcoal, and hemoperfusion for decontamination, seizure control with benzodiazepines, and management of arrhythmias.^[3,9–11,17,24]^ The specific antidote, acetamide, acts as an acetate donor, mitigating the metabolic blockade caused by fluoroacetate.^[15,19,23]^ Notably, our case suggests that the delayed administration of acetamide in the early stages may still be effective. This highlights the importance of empirically using this drug in suspected cases.

## 5. Conclusion

Acute fluoroacetamide poisoning is a severe and under-recognized condition that requires high vigilance, particularly in regions where illicit pesticides are still used. This case, along with the literature, demonstrates that complete recovery is possible with the early initiation of acetamide therapy and aggressive supportive care. Diffusion-weighted MRI is a valuable diagnostic and monitoring tool that characteristically reveals reversible leukoencephalopathy. Greater awareness and early neuroimaging can significantly enhance diagnostic accuracy and assist in guiding prompt treatment, resulting in improved outcomes in these potentially fatal poisonings.

## Author contributions

**Data curation:** Zhongliang Xu, Shaopeng Ma.

**Funding acquisition:** Zhongliang Xu, Zhengze Shen.

**Investigation:** Shaopeng Ma.

**Formal analysis:** Zhuangfei Li, Shengkun Yang.

**Project administration:** Deyu Yang.

**Supervision:** Zhengze Shen.

**Conceptualization:** Shudong Liu.

**Resources:** Shudong Liu.

**Writing – original draft:** Zhongliang Xu, Shaopeng Ma.

**Writing – review & editing:** Shudong Liu.
